# Specimen Size and Environmental Exposure Effects on Initial Diffusion in E-Glass/Vinylester Pultruded Composites

**DOI:** 10.3390/polym17060815

**Published:** 2025-03-20

**Authors:** Vistasp M. Karbhari

**Affiliations:** 1 Department of Civil Engineering, University of Texas Arlington, Arlington, TX 76006, USA; vkarbhari@uta.edu; 2 Department of Mechanical and Aerospace Engineering, University of Texas Arlington, Arlington, TX 76006, USA

**Keywords:** pultrusion, E-glass, vinylester, composite, moisture, uptake, Fickian, diffusion, specimen size, surface, edge

## Abstract

This paper studies the effect of specimen size on the moisture uptake characteristics of pultruded E-glass/vinylester composites exposed to conditions of immersion and 99% RH over a range of temperatures. Four different specimen sizes representative of sizes commonly used for material characterization (tension, short-beam-shear, and dynamic mechanical thermal analysis) as well as moisture uptake are included. It is shown that both exposure conditions and geometry significantly influence uptake behavior, and that the differences, in general, can be elucidated through consideration of surface-to-edge area ratios of the specimens. For the current study, the ratio extends from 2.528 at the lowest level for the short-beam-shear specimens to 16.979 at the highest for the tensile specimens. The overall levels of uptake in the period of exposure, the levels of transition uptake, and the diffusion coefficients are noted to increase with a decrease in the ratio, suggesting an increased influence of the edge effect, which is further enhanced with an increase in temperature. Levels of normalized transition uptake for the specimens with the lowest surface-to-edge area ratio are 12.5 and 8.2 times higher than those for the specimens with the highest ratio at the two extreme temperatures, respectively, when exposed to 99% RH, and are 7.2 and 15.3 times, respectively, under conditions of immersion. Activation energy calculations also highlight differences based on specimen size and the condition of exposure with immersion leading to a lower activation energy than exposure to 99% RH when considering the initial linear regime with the specimens having the largest surface-to-edge area ratios showing 11.3–13.5% higher levels due to exposure to 99% RH, whereas the two specimens with the smaller ratios show a 4.9% increase. The findings highlight the importance of specimen size and exposure conditions and emphasize that the commonly used assumptions could lead to inaccurate results especially when extrapolated. The use of the immersion condition as a means of accelerating field conditions of humidity could significantly overestimate effects. Further, the direct use of uptake characteristics from specimens at one size, or surface-to-edge area ratio, could lead to inaccurate conclusions if extrapolated to specimens that are significantly different leading to design and durability prediction implications.

## 1. Introduction

The growing demand for lightweight, high-performance profiles, and panels, for use in the civil, offshore, and marine infrastructure sectors that also require corrosion resistance and low maintenance in harsh and changing environmental conditions at low cost has led to the increased consideration and use of fiber-reinforced polymer (FRP) composites fabricated using the pultrusion process [1,2]. There is also increasing use of these systems in power and transmission applications where resistance to heat damage, thermal warping, and electrical arcing provide significant advantages over conventional materials [3,4] as well as in the growing wind turbine sector, where high specific stiffness, tailored properties, high durability, and lightweight characteristics are of specific importance [5,6]. While there is a history of successful field implementation with these materials, there is still a lack of in-depth understanding of moisture uptake kinetics and hence of the comprehensive long-term durability of these materials compared to those of metals, or autoclave cure composites, and these data are even further limited as related to vinylester based glass fiber-reinforced pultruded composites [7,8]. In all these applications, exposure to moisture in the form of humidity and solution represents a significant factor in environmental durability, and hence the development of a comprehensive understanding of kinetics of moisture uptake, and the consequent effects of this are of critical importance. Recent reviews of models of moisture uptake [9] and of effects of hydrothermal aging [7] elucidate these aspects, and hence, further details will not be repeated herein.

In general, exposure to moisture leads to the filling of free volume, followed by the interaction of water molecules with polar groups of the polymer network leading to a transition from absorption to adsorption, which can lead to swelling and penetration into cross-linked regions [10] as well as interface debonding resulting in increased uptake through capillary action at the fiber matrix interface. Moisture can lead to hydrolysis of ester groups in the resin resulting in the formation of carboxyl groups that increase deterioration [11], and can further result in plasticization, which can cause depression of the glass transition temperature and decreases in mechanical performance characteristics [12] as well as hydrolysis, which can lead to additional degradation of the resin, matrix microcracking, and further matrix fiber matrix debonding [13,14]. These paths and mechanisms lead to more rapid uptake and deterioration than would normally occur by molecular diffusion through the bulk polymer [14]. In addition, longer-term exposure can lead to leaching of low-molecular-weight species, and even filler particles, leading to further degradation [7,15,16,17]. Thus, the development of a comprehensive understanding of the rates and levels of moisture uptake and the consequent phenomena is critical for prediction of long-term durability of these materials. However, the data are often lacking due to the range of material systems and conditions possible, with published data often reporting erroneous predictions since diffusion rates themselves have been reported to vary by factors of 10 to 30, or more, depending on details of materials and exposure condition [18].

Fickian diffusion models, which intrinsically assume the attainment of an equilibrium level of moisture uptake representative of saturation with diffusion driven entirely by the concentration gradient, are commonly used to describe uptake in polymers and composites. Uptake is often realized as a two- or multi-stage/phase phenomenon with both concentration-gradient-driven diffusion over the short term and time- and moisture-uptake-dependent relaxation and deterioration over the longer term [19,20,21]. Deviations in uptake response can be related to polymer structure and exposure condition [22], with even saturation levels changing with aspects such as humidity level [23], with the attribution of temperature of exposure as the driver of the second step being debated [23,24]. Since the initial stage is largely linear and diffusion dominated [25], Fick’s law is often used to characterize overall response even though non-Fickian response is exhibited over extended periods of time [26]. It should be noted that even here, diffusion mechanisms and rates are dependent on conditions of exposure such as temperature and relative humidity [27], type of solution to which the material is exposed [28,29,30], reinforcement details such as fiber volume fraction, fabric type, and orientation [31,32,33,34,35], as well as degree of cure attained prior to exposure [36]. Further, even in the initial regime, polymer level interactions and increased fiber-matrix debonding, and subsequent wicking, can lead to complex interactions and consequent rate changes. There are also differences that can be attributed to the method of testing including the size of specimens and whether edges are sealed [35]. While both ASTM D5229/D5229-M [37] and ISO 62:2008 [38] provide guidance on specimen size for the determination of diffusivity constants, these are often not practical, and other sizes have been extensively reported in the literature with a smaller size of about 25 mm by 25 mm being commonly used for determination of diffusion coefficients [7,34,39,40,41]. It should be noted that while ASTM D5229/D5229-M [37] mentions the desirability of a nominal length to thickness ratio of 100:1, presumably to minimize edge effects, this ratio is often not practical for the purposes of laboratory, or even field, testing, and may not be representative of conditions of use of composites in components. Notwithstanding the ambiguity related to specimen size, there are also circumstances when there is a need to determine moisture kinetics at the size level of the test specimen used for mechanical, or thermomechanical, characterization to enable a direct correlation between these characteristics and the rate and level of uptake at the point when these characteristics are determined. While a level of scaling is possible by the consideration of parameters such as $\frac{\sqrt{t}}{h}$ and $\frac{t_{sat}}{h}$ [42], where *t* and *t_sat_* are the time of exposure and time to saturation, respectively, and h is the specimen thickness, as well as the use of edge correction factors [25,43,44], these approaches have limitations including those related to differing paths of moisture transport due to anisotropy and the effects of specimens having significantly different aspect ratios, effective fiber directionality, and even the significant difference between the assumed mode of Fickian diffusion and the more complex reality, which includes increased changes in matrix network structure, the early competition between mechanisms of moisture induced post-cure and deterioration, and even capillary paths through fiber-matrix debonding [11,28,45]. The characterization of moisture uptake kinetics is extremely complex, and this has led not just to conflicting results in the published literature but also the lack of comprehensive predictive models of mechanical performance changes as a direct result of uptake. This is especially important since durability tests conducted under laboratory settings often use conditions of acceleration, which are assumed to replicate longer-term field exposure, but may not result in similar rates of uptake and/or levels of threshold, transition, and/or saturation. For example, elevated temperature immersion in water is often used to simulate longer-term exposure to humidity even though the effects of immersion and exposure to humidity may not be equivalent as related to moisture kinetics. Given the correlation between the rate and level of change in performance characteristics and rate and level of moisture uptake, it is critical to develop a comprehensive understanding of diffusion processes and changes therein as a function of period and type of exposure, in order to enable the development of phenomenological and mechanistic models for the prediction of long-term durability [10,43,46,47].

**Figure 1:**
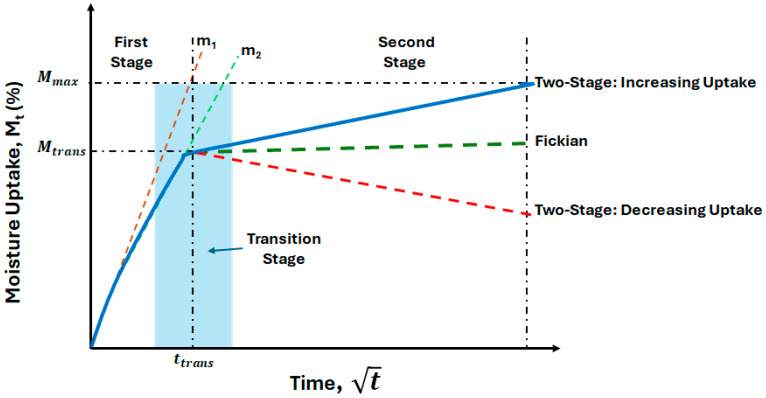
Schematic of stages in moisture uptake.

This paper focuses on the investigation of the effect of two interacting aspects on initial moisture uptake kinetics: (a) specimen size and (b) effective exposure conditions. In the current context, these are based on conditions of 99% relative humidity (RH) and immersion in deionized water over a range of temperatures. The two specific conditions were selected to assess differences between two exposures that are often treated as equivalent for purposes of mechanical property characterization after long-term exposure. Recognizing the complex nature of these phenomena, this study focuses on the initial potential period of water uptake only, enabling the approximation of Fickian response, while still acknowledging that longer-term response is non-Fickian. This is expected to provide useful insight into the first stage of uptake, as shown schematically in Figure 1. This is aimed at enabling a focus on the initial effects of exposure as part of a larger study into all three stages—diffusion-dominated, transition, and relaxation/deterioration-dominated, as depicted in Figure 1. As noted earlier, while stage I is assumed to be diffusion-dominated and stage III is indicative of effect of longer-term relaxation and deterioration [21,48], the first stage is also affected by more complex reversible and irreversible phenomena.

## 2. Materials and Methods

### 2.1. Materials and Process Details

Panels of 1.36 mm nominal thickness and 100 mm width were pultruded at a line speed of 91 cm/minute with die temperatures in the range of 120 °C. The fiber reinforcement consisted of unidirectional E-glass in continuous filament single end rovings of 4400 g/km tex with a bisphenol epoxy-vinylester resin (Reichold Dion VER 9102) and ASP 400 grade kaolin particles of 2.58 specific gravity and 4.8 mm average size. Fiber volume fraction was determined as 68% using burn-off test procedures, and the glass transition temperature, determined prior to conditioning and environmental exposure, as measured in three-point bend mode using dynamic mechanical thermal analysis (DMTA), was determined to be 117 °C. While the use of the pultrusion process results in higher glass transition temperatures and fiber volume fraction than other non-autoclave cure processes, which can both positively impact long-term durability, it must be kept in mind that vinylesters follow a complex progression of cure due to simultaneous, but rate-differentiated, homopolymerization and copolymerization reactions [49,50], which can affect moisture uptake and initial resistance to hydrolysis as well as degree of susceptibility of swelling with solvents, resulting in differences in long-term durability [11,51]. In addition, the hydrolysis of ester groups can result in the formation of carboxyls that can accelerate deterioration through autocatalysis [11].

### 2.2. Specimen Details

Specimens for assessment of moisture uptake characteristics were cut to four rectangular geometries that included sizes that are used for tensile testing, short beam shear testing, DMTA testing, and a commonly used 25.4 mm by 24.5 mm size, which is representative of specimens used for moisture uptake. Details related to these specimens are given in Table 1. All specimens were cut ensuring that they were at least 1 mm away from the edges of the pultruded panel to ensure that there were negligible effects of the die surface on cure and/or fiber and particle distribution due to the tool. All specimens were finished by wet-sanding of edges with 320-grit silicon-carbide polishing paper and were then carefully checked for surface porosity, micro cracks, and other non-uniformities/damage that might unduly affect moisture uptake. Specimens having these were rejected. Surfaces were not sealed, following the rationale provided by Korkees [35].

### 2.3. Moisture Uptake Test Procedures

Moisture uptake testing was conducted under conditions of 99% RH and through immersion in deionized water, with both conditions being at temperatures of 20°, 40°, 60°, and 80 °C for periods up to 96 weeks, and with measurements being recorded at preset intervals. A minimum of five specimens were used for each measurement period. Specimens exposed to humidity were placed in humidity chambers with relative humidities controlled within ±1% RH and temperatures maintained to within ±3 °C, which was also the range used for the baths used for immersive exposure. Specimens were arranged to assure no contact between adjacent specimens, thereby precluding differences due to overlap induced blocking of moisture in any way during the exposure period. All specimens were preconditioned at 40 °C and 18% RH for six weeks prior to exposure to provide a uniform baseline and minimize variation in post-cure and embrittlement. Test specimens were removed from exposures at periodic intervals with padded tweezers to ensure that the pressure on surfaces was minimized and that potential contamination from hands in the form of dirt and oils were minimized. Measurement of mass were made on a micro-balance with the resolution of 10^−5^ g. Apparent moisture content (reported as percentage change), *M_t_*, measured at time, *t*, is determined from

(1) $$M_{t}=\frac{M_{w}-M_{0}\;}{M_{0}}\times 100$$

where *M*_0_ is the initial mass measured after conditioning and *M_W_* is the mass determined after exposure for time *t*. Due to the complex interactions/reactions caused by exposure, as noted previously, there is simultaneous uptake and change/deterioration in the polymer and composite.

Overall uptake can generically be represented in three stages, as shown schematically in Figure 1. The first stage (Stage I) is diffusion-dominated, and the third (Stage III) represents the longer-term response that results in non-Fickian uptake, in that an equilibrium level is not attained even after extended periods of time. The transition region (Stage II) represents the region between these two and can be fairly broad but is represented by a point where behavior makes a complete shift from diffusion dominance to the longer-term relaxation/deterioration-based response. It should be noted that even in Stage I, the rate of uptake can change, with the rate being faster initially (as represented by *m*_1_ in Figure 1), followed by a lower rate that represents the overall regime (as represented by *m*_2_ in Figure 1). It is emphasized that while *m*_1_ is representative of the initial slope (the initial 60% of the initial region if Fickian response is assumed), *m*_2_ is representative of the overall behavior and hence should not be considered equivalent to the slope.

While the mechanisms of uptake initially dominate (in the diffusion dominated regime—Stage I in Figure 1), the loss can at times be greater, resulting in a decrease in overall mass recorded. In keeping with the focus of the investigation on the initial diffusion-dominated regime, analysis of moisture uptake rates and kinetics is only conducted till the attainment of a peak, and thus for specimens showing two-stage response with a decrease in uptake after attainment of a peak, we neglect changes after the peak for the purposes of the current investigation (which is focused on Stage I) with the approximation that the peak represents *M_max_*, the maximum uptake achieved due to exposure. This would result in the overall period under consideration being less than 96 weeks for those specimens where the mass change decreases after the attainment of a peak, and this was considered in subsequent analysis. This in no way suggests that mass loss only occurs after attainment of the peak, and does not occur in Stage I. Rather, the extent of loss is far lower in Stage I and is overshadowed by the level of uptake resulting in a positive rate of increase in specimen mass. It is emphasized that the focus of the current investigation is on the initial period of uptake rather than the full uptake curve, or even the full extent of Stage I uptake, and thus the mass loss in the regime under consideration, which is extremely small, is unlikely to affect the results.

## 3. Moisture Kinetics

**Figure 2:**
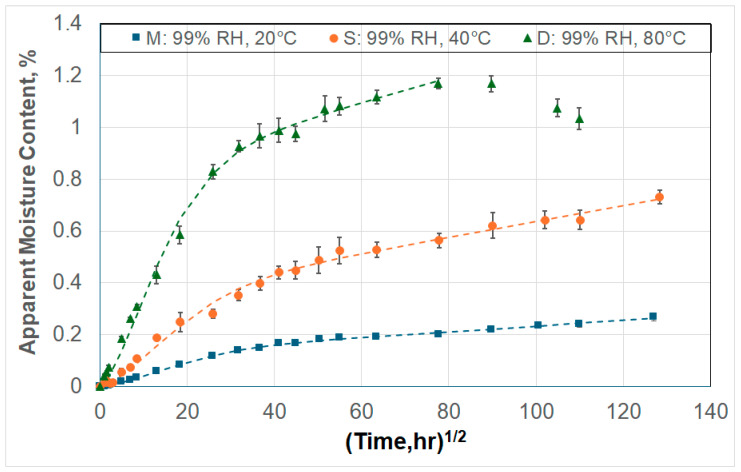
Representative moisture uptake for 3 typical specimens showing 2-stage response.

Given the focus of the current investigation, characteristics of moisture uptake were assessed with respect to Stage I uptake. To differentiate between the overall trends in Stage I and that of the initial region, which has a higher rate of uptake and is linear, consideration is given to both the initial response and the overall response, as discussed in this section. Figure 2 shows a representative set of moisture uptake curves, wherein two of the specimens do not attain a saturation level within the 96-week period of exposure and the third shows a drop in uptake after attaining a maximum level. All three depict characteristics of a two-stage response with a rate of uptake being significantly less in the second stage for the first two and being negative (i.e., representative of mass loss) for the third. All specimens included in this investigation show a response following these two types, varying based on the condition and temperature of exposure and specimen type.

The response in Figure 2 does not follow Fickian behavior, which is characterized by the attainment of an equilibrium level. Further, the response shows distinct phases/stages, including an initial linear (or close-to linear) stage, followed by a stage with a slower rate of uptake, and then for some specimens, a third stage in which mass loss dominates. A comprehensive review of different models is provided in [9], and based on the characteristics shown in the response from the set of specimens under this investigation, a two-staged model was selected. Uptake for a two-staged process can be described following [21] as

(2) $$M_{t}=M_{\infty 0}\left(1+k\sqrt{t}\right)\left\{1-exp\left[-7.3{\left(\frac{Dt}{h^{2}}\right)}^{0.75}\right]\right\}$$

where *M_∞_*_0_ and *D* are the equilibrium moisture uptake level and diffusion coefficient associated with stage I uptake, and k is a time dependent coefficient characteristic of the rate of polymer relaxation and second stage damage due to the moisture absorbed. When *k* = 0, Equation (2) reverts to the Fickian form of diffusion. For the purposes of the current investigation, the parameters *D*, *k*, and *M_∞_*_0_ were determined by a best fit of Equation (2) using the least difference of squares. For purposes of comparison with Fickian response, the parameter *M_∞_*_0_ in Equation (2) represents the transition point, *M_trans_*, between Stage I and Stage III response, which is taken to be the equilibrium point for the hypothetical Fickian response. This method compares well for the determination of the transition point, *M_trans_*, with the process described in Korkees et al. [40] for the determination of the transition while also enabling the determination of additional overall parameters.

It is emphasized that the value of diffusivity, D, includes some of the longer-term effects since the equation provides for both the diffusion dominated and longer-term relaxation/deterioration modes, as will be discussed later. A comparison of values of *M_max_*, *M_∞_*_0_, and *D* are provided in Table 2 for the two exposure conditions at four temperature levels. As can be seen from Table 2, the level of peak moisture uptake, *M_max_*, increases with temperature in line with the previous research [7,52,53,54]. The increase in temperature increases thermal energy, resulting in an increase in segmental motion of the polymer chains, which then allows absorption into dense regions, effectively expanding the free volume. However, a comparison between levels attained due to exposure to 99% RH and immersion indicates a variation depending on temperature level and, to a minor extent, the specimen size. At the lowest temperature level of 20 °C, the uptake is higher in the case of immersion. This is attributed to direct contact with water, whereas the exposure to relative humidity does not have the same driving force for moisture transport across the specimen surface barrier. As temperature increases, however, the levels are higher for cases of exposure to relative humidity except for three sets—D specimens at 40 °C and 60 °C and T specimen at 40 °C. In all three cases, the values are fairly close and within scatter bounds. This further emphasizes that while immersion is often used as a means of assessing durability, the assumption that the conditions can be considered to be equivalent to, and that the effects of immersion are perhaps harsher than, those due to exposure to humidity may not be appropriate due to different mechanisms that cause water transport both across the surface and internally.

Given the effect of moisture uptake on performance characteristics and the value of being able to correlate uptake levels to changes in performance characteristics, it is of interest to assess the effect of specimen size on the level of maximum uptake level recorded. The values of *M_max_* in Table 2 indicate substantial variation in the levels of maximum uptake with specimen type. Further assessment is possible by consideration of the modes of moisture uptake through the consideration of the ratio of surface area to edge area (hence surface-to-edge area ratio in Table 1) of each specimen as a means of determining the relative effect of uptake that occurs through the surfaces (i.e., with moisture transport through the thickness) to that which occurs through the edge faces. For the specimens considered, the surface-to-edge area ratios are 2.527, 5.719, 9.338 and 16.979 for the S, D, M, and T specimens, respectively, indicating an increasing extent of surface area over which absorption can occur as the ratio increases. Under conditions of 99% relative humidity, M, D, and T specimens show a linear relationship between uptake and temperature, indicating surface-driven uptake dominance, whereas S specimens, which have the lowest surface-to-edge area ratio, show a distinct two-stage response of rapid initial increase in uptake with temperature dominated by edge effects followed by a slower saturation or relaxation stage. In comparison, under conditions of immersion, the direct contact with water results in greater surface diffusion, wherein larger ratioed specimens such as M and T show a linear relationship of uptake, with temperature emphasizing the dominance of surface-based uptake, whereas the smaller ratioed D and S specimens in which absorption is more edge-driven show two-stage response as a function of increase in temperature.

**Figure 3:**
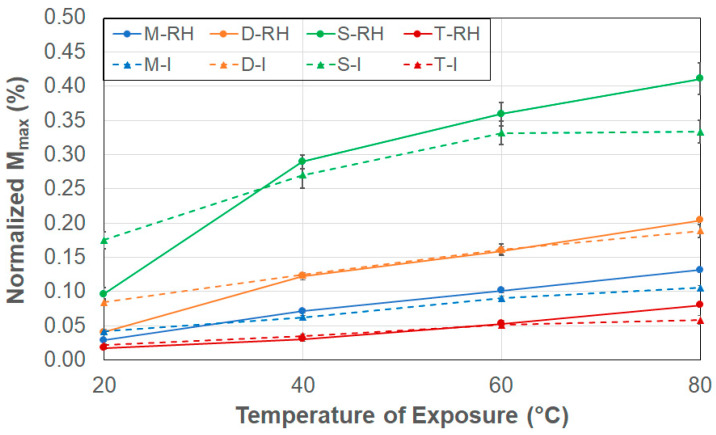
Level of Maximum Uptake normalized by the surface-to-edge area ratio as a function of exposure condition (RH: 99% RH, I: Immersion in deionized water) and temperature.

The effect of specimen size is seen more clearly through the normalization of uptake by the surface-to-edge area ratios, as shown in Figure 3, where the results are depicted in terms of averages and standard deviations. Here, a predominantly linear relationship is clearly seen for the M and the T specimens, which also show the similarity between the responses from the two exposure conditions of 99% RH and immersion, whereas a two-staged relationship indicative of change in mode can be seen with a smaller ratio D and S specimens, which have lower surface-to-edge area ratios. It is of note that there is a significant difference between the RH and immersion-based responses in the case of the S specimens, which, in fact, have the lowest surface-to-edge area ratio. The differences in uptake levels between the two conditions of exposure can be attributed to the difference in driving forces (i.e., boundary conditions) and the resulting mechanisms. Immersion results in a constant saturation condition around the specimen, whereas exposure to humidity involves vapor-phase diffusion, which is a slower and more time-dependent process. Thus, at lower temperatures, direct contact due to immersion results in higher uptake. However, at elevated temperatures (60° and 80 °C), mechanisms of polymer relaxation and water–polymer chain interactions dominate due to moisture-induced relaxation. This difference, which will be further discussed later as related to other parameters as well, suggests that the assumption of immersion being an accurate substitute for humidity conditions seen in the field could lead to incorrect, or at least highly exaggerated, predictions of deterioration in long-term performance. For both exposure cases, uptake levels at all temperatures clearly show the effect of the ratio, with the level of uptake increasing in the order T < M < D < S, which is in line with the decrease in the surface-to-edge area ratios, emphasizing again the difference in direction of uptake with size, with larger specimens having dominance of uptake due to surface effects, while smaller specimens show greater influence of uptake due to edge effects, especially at lower temperatures.

As shown in Figure 1, the transition point, *M_trans_*, indicates the change in response from a predominantly diffusion dominated mode to the slower, and longer-term, relaxation/deterioration mode. This point is also indicative of the end of Fickian response and can be considered, following [43,52], equivalent to the Fickian equilibrium uptake level. As can be seen from Table 2, the level of *M_trans_* increases with temperature for specimens exposed to conditions of 99% RH. This aligns well with the expectation that exposure to higher temperatures accelerates diffusion, leading to higher levels of uptake including the equilibrium point. However, under conditions of immersion, the response is markedly different, showing an increase from 20 °C to 40 °C, followed by a decrease at 60 °C, and then an increase again to 80 °C, suggesting a change in mechanisms within the upper range, as noted previously by Bao and Yee [43]. Trends based directly on size are mixed. Under conditions of relative humidity and immersion, the smallest sized specimen, S, showed the highest level of transition uptake at all levels except 60 °C under conditions of 99% relative humidity, whereas the lowest levels were shown in general by the largest sized specimen, T, at both 20 °C and 40 °C under conditions of 99% RH and 40 °C, 60 °C and 80 °C under conditions of immersion. This is generally in line with the results reported by Grammatikos et al. [54], who, based on experiments related to three different sized square specimens, reported that equilibrium level decreased as the size of the specimens increased, which was in congruence with an increase in the surface-to-edge area ratio, termed the veil area edge ratio in [54].

**Figure 4:**
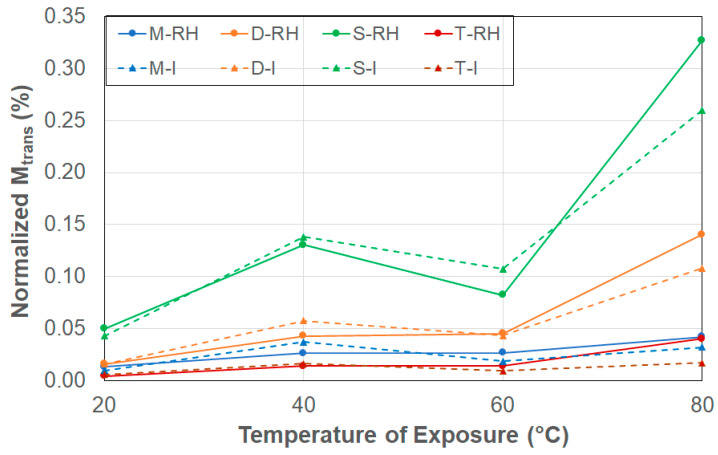
Level of normalized *M_trans_* as a function of exposure condition (RH: 99% RH, I: Immersion in deionized water) and temperature. Results are depicted in terms of averages only since the level of standard deviations is too low to differentiate.

As in the previous case, the effect of size is highlighted better through the consideration of levels normalized by the respective surface-to-edge area ratios (=*M_trans_*/Surface-to-edge area ratio). In all cases, the level of normalized *M_trans_* is seen to increase with the size of specimens, with the specimen having the largest ratios, T and M, having significantly lower levels than the smaller specimens, D and S, with the T and S specimens showing the lowest and highest levels of normalized *M_trans_*, respectively. Overall response can be seen in Figure 4, where the change in trends is clearly seen for all specimens and both conditions at 60 °C. The drop in *M_trans_* at 60 °C suggests a transition in moisture update mechanisms related to competition between acceleration of diffusion due to micro-cracking and wicking, as well as changes in polymer morphology, and due to polymer relaxation effects. It is hypothesized that polymer-free volume could reach a critical threshold at this temperature, enabling greater adsorption at the molecular level, resulting in volumetric expansion of the bulk polymer due to increased susceptibility to swelling as reported by Apicella at al. [51], reducing the deterioration-induced capillary intake at the debonded fiber–matrix interfaces. Further, hydrogen bonding between the water molecules and polymer chains can result in localized traps of water for finite periods that could hinder uptake [55]. Ramesh et al. [56] also reported complex mechanisms of solvent diffusion based on temperature and concentration interactions resulting in competition between free volume trapped in the polymer at temperatures below the glass transition temperature that can be continuously redistributed and the slower relaxation process in conjunction with plasticization that can cause hindrance in the filling of free volume. Specimens with lower surface-to-edge area ratios correspond to a higher edge area exposure, which accelerates moisture uptake, with the effect being more pronounced at higher temperatures, where both diffusion and damage mechanisms can be expected to be enhanced. In contrast, the larger surface-to-edge area ratio specimens have a greater bulk that effectively retards the uptake, thereby effectively limiting the edge effect. As can be seen in Figure 4, the two smaller sized specimens show elevated increase in uptake at the highest temperature of exposure, with the level being a bit lower in the case of immersion than due to 99% RH, again emphasizing size effects.

*M_max_* and *M_trans_* represent two important characteristics of moisture uptake indicative of key levels attained during a test. Given the importance of the two regimes, and especially in the context of the initial diffusion-dominated regime, which is the subject of this paper, it is of interest to assess the influence and extent of this regime. This can be undertaken through the assessment of the ratio $\frac{M_{trans}}{M_{max}}$, with higher values of the ratio indicative of a greater relative extent of the initial regime across the entire moisture uptake profile.

Table 3 shows the extent of the initial regime as a percentage of the maximum moisture uptake from which it can be seen that at the lowest temperature level in the case of exposure to 99% RH specimens with the lowest surface-to-edge area ratio, S, have the highest extent of 51.66% (i.e., the level of *M_trans_* is 51.66% that of the maximum uptake level), whereas the specimen with the highest surface-to-edge area ratio, T, has the lowest extent of 24.66%. It is of interest to note that with the exception of the specimens with the highest surface area to edge ratio, T, all specimens show a near-linear decrease in extent of the diffusion dominated regime from 20° to 60 °C, showing earlier attainment of the transition threshold, followed by an increase, the extent of which is greatest for the two smallest specimens, S and D. Between 60 °C and 80 °C, the S specimens (with the lowest surface-to-edge area ratio of 2.528) show an increase from 22.91% to 79.65% of the maximum uptake, an increase of 56.74, with the next lowest—the D specimens (with the surface to area ratio of 5.719)—showing an increase of 40.27. In comparison, the M specimens show a very minor increase from 26.12% to 32.10%, i.e., an increase of just 5.98 over the same temperature range. In contrast, the T specimens, which have the largest surface-to-edge ratio, show an increase equivalent to almost double the extent from 20 °C to 40 °C, followed by a decrease of about the same extent from 40 °C to 60 °C, followed by a larger increase. This response can be correlated with changes in mechanisms from surface to edge dominance, as described earlier for uptake. Given that the specimens placed in the immersion environment all have more direct transport through surfaces, it is noteworthy that the same trends are shown by all specimens immersed in water, suggesting that immersion in fact has the effect of increasing the extent of uptake through surfaces, as is also noted by specimens with the highest surface-to-edge area ratios exposed to 99% RH. It should also be noted that the highest extent of the diffusion dominated regime under both exposure conditions at 80 °C is shown by the S specimens which have the lowest surface-to-edge area ratios reflecting greater edge driven behavior and slower saturation at this temperature while the lowest extent is shown by the M specimens under conditions of 99% RH, and T specimens under conditions of immersion, again indicating faster transition due to bulk effects. The effect of acceleration of aging, which is often modeled through an increase in temperature of exposure, should thus be carefully reviewed, since, as discussed above, effects can be significantly influenced by specimen size, and hence results of uptake at one size should not be directly used for predictions on specimens of very different sizes, or at least with very different surface-to-edge area ratios.

As seen in Table 2, the values of diffusion coefficients increase with temperature for the two specimens with the larger surface-to-edge area ratios, M and T, as a result of exposure to both 99% RH and immersion conditions, with the values from immersion being higher. This is in line with the expectation that direct contact with water, as in immersion, would lead to higher rates of transport within the composite. In contrast, the smaller specimens, D and S, show significant variation with temperature, suggesting competing effects and different dominant modes with changes in temperature and edge effects. At the lowest temperature, under both conditions of exposure, the smaller specimens exhibit the highest values of diffusion coefficients, which can be attributed to edge effects dominating, wherein moisture penetrates material faster at smaller sizes due to higher surface area to volume ratios. When diffusion coefficients are normalized by the surface-to-edge area ratios, the lowest normalized coefficient values are indicated by the specimens with the highest surface-to-edge area ratios for all temperatures and for both conditions of exposure. In comparison, the highest values are indicated by the smallest specimens, S, which have the lowest surface-to-edge area ratios across all temperatures for immersion and at the two highest temperatures in the case of exposure to 99% RH with the D specimens, which have the next lowest surface-to-edge area ratios, having the highest normalized diffusion coefficients at the two lower temperatures.

**Figure 5:**
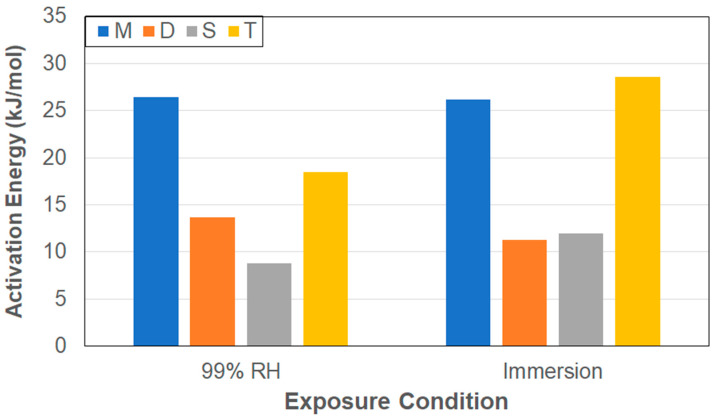
Activation energy for first-stage diffusion as a function of exposure condition and specimen type.

Given the trends indicated as a function of both specimen size and exposure conditions, it is of interest to determine the activation energy that must be overcome for moisture uptake, as represented by the first stage diffusion parameter using an Arrhenius-type relationship [57] as

(3) $$D=D_{0}\;exp\left(\frac{-E_{a}}{RT}\right)$$

where *D*_0_ is a temperature-independent constant, *R* is the universal gas constant (8.3143 J/mol K) and *T* is the temperature on the Kelvin scale. Plotting *ln* (*D*) versus 1/*T*, values of the activation energy, *E_a_*, for the first stage diffusion can be determined and are shown in Figure 5 for two exposure conditions and four specimen types. The larger specimens show higher levels, and the smaller ones significantly lower ones. It is noted that the activation energies for the larger specimens are in the range of those reported previously for pultruded E-glass vinylester composites of 26.04 kJ/mol [58] and 20.45 kJ/mol [59], whereas specimens D and S exhibit values closer to those of wet layup E-glass vinylester with lower fiber volume fractions of 12.62 kJ/mol [60] and 11.72 kJ/mol [61], which is similar to that of unreinforced vinylester reported as 12.14 kJ/mol [59]. The lower values of activation energy for the smaller specimens suggests that the barrier to transport into the bulk may be effectively reduced by the larger role played by the edge surfaces resulting in faster transition through greater capillary transport as a result of fiber–matrix debonding, and that these specimens may be consequently also subject to earlier effective matrix relaxation and deterioration. It should be emphasized that the diffusion coefficients were determined using the two-stage model [21], which considers both diffusion- and relaxation-dominated stages but does not completely isolate the two [9], thereby resulting in the apparent diffusion coefficient for the smaller specimens being affected to a larger extent by matrix effects such as relaxation and deterioration as well as fiber matrix debonding, which would lead to differences in initial uptake, especially at later stages of the diffusion dominated process, but still prior to the attainment of *M_trans_*.

To further isolate effects of the diffusion-dominated regime on the determination of the diffusion coefficient, the initial slope of the uptake curve till 60% of *M_trans_* (representing the 60% level of *M_∞_* that would conventionally be used for determination of the slope under Fickian uptake [25,27,62]) was used to determine the diffusion coefficient, *D_i_*, representative of the very initial state of uptake (see Figure 1) as

(4) $$D_{i}=\frac{\pi m^{2}h^{2}}{16M_{\infty }}$$

where *m* is the initial slope, and *M_∞_* is the level equivalent to that attained through Fickian equilibrium, which is taken to be *M_trans_*. It should be noted that this effectively considers only the very initial portion of Stage I, as shown in Figure 1, further restricting analysis to a regime of uptake which is more likely to be based on diffusion phenomena alone rather than other additive processes. Chateauminois et al. [14] emphasized that in the early stages, moisture uptake increases linearly and can be considered to remain in a single free phase, driven to penetrate the bulk resin only through the water concentration gradient.

Values of these diffusion coefficients are reported in Table 4. A comparison with the values of the diffusion coefficient from the two-stage model clearly shows that the ones determined using Equation (4) are higher, emphasizing that they more closely represent the diffusion-based phenomena seen in the early stages of uptake where sorption is expected to be rapid, whereas the coefficients from the two-stage model effectively aggregate response over the entire first stage, which includes aspects related to slower phenomena including those related to irreversible changes in polymer chain morphology as well as the initiation of longer state, slower relaxation and deterioration phenomena in the transition region prior to attainment of *M_trans_*. The differences highlight what is already shown schematically in Figure 1 through the lines *m*_1_ and *m*_2_, indicative of the two aspects considered here. It can be seen from Table 4 that the diffusion coefficients related to initial uptake increase with temperature for all specimens under both exposure conditions, with the immersive conditions resulting in a higher, i.e., faster, rate of uptake at almost all levels. The exceptions to this are the M and T specimens at 60 °C, both of which show changes in the initial slope very early potentially due to changes in mechanisms of localized uptake. The higher values due to immersion emphasize the greater driving force that exists when water is in direct contact with specimens, which is borne out through the determination of activation energies for the two conditions using the Arrhenius equation in Equation (3) using values of *D_i_* rather than *D*.

**Figure 6:**
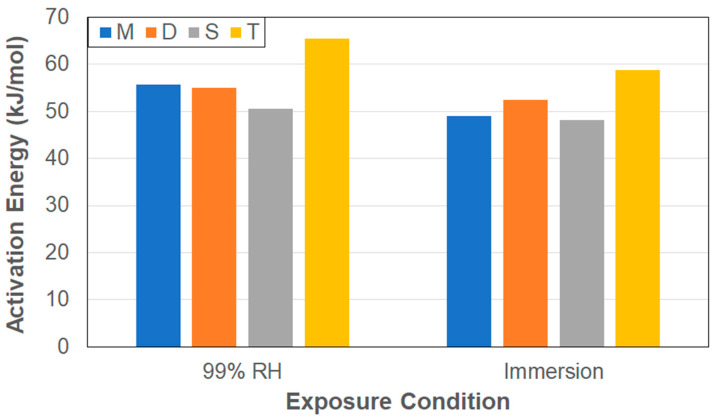
Activation Energy for initial diffusion as a function of exposure condition and specimen type.

As expected, as shown in Figure 6, the values of the activation energies are lower in the case of immersion due to the greater driving force from the saturation boundary conditions. It is of interest to note that the highest value for each condition is shown by the T specimens, which have the greatest surface-to-edge area ratios, and the lowest by the S specimens, which have the lowest surface-to-edge area ratios, again indicating the difference in response due to specimen size and effects of surfaces versus those of edges. The initial diffusion coefficients also show the same tendencies, with the general order from lowest value to the highest being T, M, D, and S, which is in the reverse order of surface-to-edge area ratios, further emphasizing the effect of size and the modes of surface and edge diffusion.

The results clearly show the differences accruing from specimen size and surface-to-edge area ratios, emphasizing that it is not reasonable to assume that diffusion occurs only in the thickness direction or that edge effects can be neglected. In fact, based on the exposure condition and specimen dimensions, the opposite is shown to be relevant. It is thus of interest to determine the principal diffusion coefficients, parallel and perpendicular to the fiber direction, respectively, for each of the exposure conditions, and thus further assess the difference between exposure to a level of 99% RH and that of immersion in water. Shen and Springer [25] reported that three-dimensional moisture uptake within the linear part of the Fickian curve could be approximated by

(5) $$D=D_{x}{\left(\frac{h}{l}\sqrt{\frac{D_{y}}{D_{z}}}+\frac{h}{w}\sqrt{\frac{D_{x}}{D_{z}}+1}\right)}^{2}$$

where *D_x_*, *D_y_*, and *D_z_* are coefficients of diffusion in the *x*, *y*, and *z* directions, respectively. In the case of unidirectional composites *D_x_* = *D_z_*, and *D_y_* is the coefficient in the direction of the fiber, with *h*, *w*, and *l* being the specimen thickness, dimension perpendicular to the fiber direction, and dimension in the fiber direction, respectively, so that Equation (5) can be expressed as

(6) $$\sqrt{D}=\sqrt{D_{x}\;}\left(1+\frac{h}{b}\right)+\frac{h}{l}\sqrt{D_{y}}$$

Using specimens of different lengths but constant thickness and width, i.e., M and T specimens, a plot of the experimentally determined initial diffusion coefficient $\sqrt{D_{i}\;}$ versus *h/l* yields a straight line with slope $\sqrt{D_{y}\;}$ and intercept $\sqrt{D_{x}\;}\left(1+\frac{h}{b}\right)$ enabling determination of the two principal coefficients. Once determined, these can then be used to re-estimate the bulk diffusion coefficients for all specimens based on the principal coefficients *D_x_* and *D_y_* determined through Equation (5), as listed in Table 5.

As can be seen, the bulk diffusivities so calculated all increase with temperature with the values due to immersion being higher than that due to exposure to 99% relative humidity. While the values for the M and T specimens are close to those reported in Table 4, as would be expected, since these were used to determine the principal coefficients, it should be noted that the values for the D and S specimens vary with values being lower at the lower temperatures and higher at the two higher temperatures, again emphasizing the difference based on edge and surface effects associated with specimen size (and surface-to-edge area ratio). It is of note that the lowest values continue to be for the T specimens, which have the highest surface-to-edge area ratios, and the highest values are from the S specimens, which have the lowest surface-to-edge area ratios, emphasizing the distinct role of specimen size and transition of dominance in moisture uptake from one based on surfaces, as generally assumed for most models, to the more complex edge-based phenomena, which include capillarity along fiber matrix interfaces. This further reinforces the earlier finding by Grammatikos et al. [54] and Cervenka et al. [63] that diffusion coefficients determined using a Fickian formula vary with sample size and that particular care must be taken in applying values of diffusion characteristics determined at one specimen size to effects at very different sized specimens.

## 4. Summary and Conclusions

The durability of fiber-reinforced polymer composites is strongly influenced by the level of moisture uptake and effects of the moisture at the levels of fiber, fiber–matrix interface, and the bulk matrix regions. This study provides information related to effects of specimen size on uptake characteristics within the initial diffusion-dominated regime, acknowledging that the overall uptake in the E-glass/vinylester composite is represented by two-stage response with a transition between the diffusion-dominated regime, which shows a more rapid rate of absorption, and the longer-term, slower, relaxation/deterioration dominated regime. For the purposes of analysis of the initial regime, the transition point, *M_trans_*, is assumed to represent the Fickian analog of the equilibrium level of uptake. The four specimens represent sizes used for the characterization of tensile (T), short-beam-shear (S), DMTA (D), and moisture uptake (M) responses under conditions of immersion in deionized water and exposure to 99% RH, both at temperatures of 20°, 40°, 60°, and 80° C. To assess the effects of size based on the mechanisms of surface and edge-based diffusion, characteristics are normalized by the surface-to-edge area ratio, where a higher value would represent greater effect of surfaces and a lower one a greater effect of edges. It is seen that both the exposure condition and specimen size significantly influence moisture uptake, with the surface-to-edge area ratio being a critical parameter to elucidate the differences. The major conclusions include the following:

- Peak moisture uptake levels, *M_max_*, increase with temperature, with levels being higher under the case of immersion at the lowest temperature of immersion, and higher under conditions of 99% RH at the higher temperatures.
- Moisture uptake levels are noted to increase as the surface-to-edge area ratio decreases, indicating that edge diffusion and capillary action at the fiber–matrix interface play a dominant role in smaller specimens in contrast to larger specimens where surface induced through-the-thickness bulk diffusion dominates.
- The transition moisture uptake level, *M_trans_*, increases with temperature, but immersion conditions exhibit non-monotonic trends, suggesting a shift in the dominant transport mechanism beyond 60 °C, where polymer swelling, microcracking, and matrix relaxation lead to deviations from simple Fickian diffusion.
- Diffusion coefficients determined using a two-stage model show an increase with temperature for the two specimens with the larger surface-to-edge area ratios, M and T, as a result of exposure to both 99% RH and immersion conditions, with the values from immersion being higher. In contrast, the smaller specimens, D and S, show significant variation with temperature, suggesting competing effects and different dominant modes with changes in temperature and edge effects. When diffusion coefficients are normalized by the surface-to-edge area ratios, the lowest normalized coefficient values are indicated by the specimens with the highest surface-to-edge area ratios for all temperatures and for both conditions of exposure.
- Diffusion coefficients derived from initial uptake rates, *D_i_*, assuming Fickian responses over 60% of the regime prior to attainment of *M_trans_* show that immersion results in faster initial uptake due to higher driving forces resulting from saturation boundary conditions. The values of the activation energies are lower in the case of immersion due to the greater driving force from the saturation boundary conditions. The highest value for each condition is shown by the T specimens, which have the greatest surface-to-edge area ratios, and the lowest by the S specimens, which have the lowest surface-to-edge area ratios.
- Values of the activation energies are lower in the case of immersion due to the greater driving force from the saturation boundary conditions. Further, smaller specimens are noted to require significantly less energy for moisture ingress than larger specimens.

The results clearly show the differences accruing from specimen size and surface-to-edge area ratios emphasizing that it is not reasonable to assume that diffusion occurs only in the thickness direction or that edge effects can be neglected. In fact, based on the exposure condition and specimen dimensions, the opposite is shown to be relevant, emphasizing that extrapolation of moisture uptake data from small specimens to larger structural components without considering surface-to-edge ratios can lead to inaccurate predictions of long-term durability. In addition, the comparison of characteristics from immersion and exposure to 99% RH indicate substantial differences, further emphasizing that while immersion is often used as a means of assessing long-term durability, the assumption that the conditions can be considered to be equivalent to, and that effects of immersion are perhaps harsher than, those due to exposure to humidity may not be appropriate due to different mechanisms that cause water transport both across the surface and internally.

## Figures and Tables

**Table 1:** Details of specimen type and geometry (* represents the fiber direction).

Specimen Type	Symbol	Dimensions (mm)			Surface-to-Edge Area Ratio
		l *	w	h	
Moisture	M	25.4	25.4	1.36	9.338
DMTA	D	10	35	1.36	5.79
Short-beam-shear	S	11	5	1.36	2.528
Tension	T	254	25.4	1.36	16.979

**Table 2:** Moisture uptake characteristics.

Characteristic	Specimen Type	99% Relative Humidity				Immersion in Deionized Water			
		20 °C	40 °C	60 °C	80 °C	20 °C	40 °C	60 °C	80 °C
*M_max_* (%)	M	0.2688	0.6658	0.9481	1.2288	0.3932	0.5856	0.8455	0.9886
	D	0.2312	0.7003	0.9103	1.1690	0.4834	0.7121	0.9235	1.0796
	S	0.2436	0.7320	0.9076	1.0376	0.4426	0.6823	0.8388	0.8432
	T	0.3026	0.5198	0.9007	1.3616	0.3725	0.6042	0.8675	0.9896
*M_trans_* (%)	M	0.1240	0.2445	0.2477	0.3945	0.0919	0.3458	0.1733	0.2980
	D	0.0899	0.2453	0.2592	0.8037	0.0908	0.3283	0.2474	0.6181
	S	0.1258	0.3300	0.2080	0.8264	0.1084	0.3494	0.2714	0.6572
	T	0.0746	0.2377	0.2353	0.6844	0.0999	0.2725	0.1596	0.2869
*D* (×10^−6^ mm^2^/s)	M	0.097	0.111	0.277	0.574	0.168	0.197	0.584	0.899
	D	0.523	0.245	0.413	0.151	0.270	0.111	0.441	0.424
	S	0.183	0.099	0.806	0.173	0.250	0.123	0.493	0.410
	T	0.094	0.099	0.254	0.289	0.106	0.160	0.396	0.728

**Table 3:** Extent of Stage I regime as a percentage of the maximum moisture content level represented through $\frac{M_{trans}}{M_{max}}$ as a percentage with higher values of the ratio indicative of greater relative extent of the initial regime across the entire moisture uptake profile.

Specimen Type	99% Relative Humidity				Immersion in Deionized Water			
	20 °C	40 °C	60 °C	80 °C	20 °C	40 °C	60 °C	80 °C
M	46.12%	36.73%	26.12%	32.10%	23.38%	59.06%	20.50%	30.14%
D	38.87%	35.03%	28.48%	68.75%	18.79%	46.10%	26.79%	57.25%
S	51.66%	45.08%	22.91%	79.65%	24.49%	51.20%	32.36%	77.94%
T	24.66%	45.73%	26.12%	50.26%	26.82%	45.10%	18.40%	29.00%

**Table 4:** Diffusion coefficients pertaining to the initial linear portion of Stage I uptake.

Characteristic	Specimen Type	99% Relative Humidity				Immersion in Deionized Water			
		20 °C	40 °C	60 °C	80 °C	20 °C	40 °C	60 °C	80 °C
D (×10^−6^ mm^2^/s)	M	3.80	19.63	45.62	188.69	5.52	37.94	47.78	225.65
	D	5.11	28.27	89.08	243.28	9.08	39.59	86.59	411.87
	S	8.30	33.18	103.87	283.53	13.20	44.18	105.68	420.91
	T	1.43	13.53	37.39	158.00	2.41	28.27	37.39	202.32

**Table 5:** Diffusion coefficients estimated from principal coefficients.

Characteristic	Specimen Type	99% Relative Humidity				Immersion in Deionized Water			
		20 °C	40 °C	60 °C	80 °C	20 °C	40 °C	60 °C	80 °C
*D* (×10^−6^ mm^2^/s)	M	3.75	19.67	45.05	187.57	5.71	37.66	47.46	224.66
	D	10.28	32.16	59.68	240.82	13.96	56.36	66.81	261.18
	S	10.61	39.38	79.01	322.68	14.93	71.27	86.42	363.90
	T	1.40	13.57	36.86	156.99	2.52	28.02	37.07	201.32

## Data Availability

The original contributions presented in this study are included in the article. Further inquiries can be directed to the corresponding author.
